# Changes in the intestinal microbiota of individuals with non-alcoholic fatty liver disease based on sequencing: An updated systematic review and meta-analysis

**DOI:** 10.1371/journal.pone.0299946

**Published:** 2024-03-28

**Authors:** Wenpin Cai, Ting Qiu, Weitao Hu, Taiyong Fang

**Affiliations:** Department of Gastroenterology, The Second Affiliated Hospital of Fujian Medical University, Quanzhou, China; University of California Los Angeles, UNITED STATES

## Abstract

**Background:**

Alterations in the composition and abundance of the intestinal microbiota occur in non-alcoholic fatty liver disease (NAFLD). However, the results are inconsistent because of differences in the study design, subject area, and sequencing methodology. In this study, we compared the diversity and abundance of the intestinal microbiota of patients with NAFLD and healthy individuals through a systematic review and meta-analysis.

**Methods:**

Three databases (PubMed, EMBASE, and Cochrane Library) were searched from their inception to March 20, 2023. A meta-analysis was performed using Stata software to analyze variations in the richness and abundance of the intestinal microbiota in patients with NAFLD. The Newcastle-Ottawa Quality Assessment Scale (NOS) was used for quality assessment.

**Results:**

A total of 28 articles were included. Shannon diversity was reduced in patients with NAFLD (SMD = -0.24 (95% CI -0.43–0.05, I^2^ = 71.7%). The relative abundance of *Ruminococcus*, *Faecalibacterium*, and *Coprococcus* all decreased, with total SMDs of -0.96 (95% CI -1.29 to -0.63, I^2^ = 4.8%), -1.13 (95% CI -2.07 to -0.19, I^2^ = 80.5%), and -1.66 (95% CI -3.04 to -0.28, I^2^ = 91.5%). *Escherichia* was increased in individuals with NAFLD (SMD = 1.78, 95% CI 0.12 to 3.45, I^2^ = 94.4%).

**Conclusion:**

Increasing the species diversity and altering the abundance of specific gut microbiota, including *Coprococcus*, *Faecalibacterium*, *Ruminococcus*, and *Escherichia*, may be beneficial for improving NAFLD.

## Introduction

The primary characteristic of non-alcoholic fatty liver disease (NAFLD) is the accumulation of lipids in hepatocytes exceeding 5% of the liver weight in the absence of an overdose of alcohol intake [1]. A meta-analysis by Younossi [2] et al. revealed an estimated global prevalence of adult NAFLD of 30%. The highest prevalence of 44% has been reported in Latin America, 31%, followed by North America, 34%, South Asia, 33%, Southeast Asia, 30%, East Asia, and 25% in Western Europe [2]. NAFLD has become a global public health problem that poses an enormous economic burden and health threat worldwide [3].

To date, the pathogenesis of NAFLD has not been fully understood. Various risk factors for NAFLD include race, genetics, and diet, with the gut microbiota and metabolites also playing important roles [4].

Recently, the theory of the "gut-liver" axis has been investigated more and more deeply. Altered dietary structure, NAFLD itself, and its complications can induce intestinal ecological dysregulation, including structural disorders of the gut microbiota, ecological dysregulation of intestinal-derived metabolites, and disruption of intestinal barriers, which further contribute to the deterioration of NAFLD [5, 6]. The correlation between NAFLD and the gut microbiota has become an important research topic, and altering the gut microbiota has become an important research direction for the treatment of NAFLD [7].

Until the current study, only one meta-analysis had investigated the composition of the gut microbiota in patients with NAFLD. Li [8] et al. included 15 studies from eight countries to compare the relative abundances of 14 gut microbiota at the genus level between patients with NAFLD and healthy individuals. Over the past two years, a growing number of researchers have analyzed variations in the abundance of microbial profiles using sequencing techniques, and numerous high-quality studies have been published on this topic. However, specific changes in the gut microbiota remain controversial and uncertain. Such new studies have inspired our enthusiasm to update the existing evidence. Therefore, this systematic review and meta-analysis summarizes and updates the changes in the gut microbiota in NAFLD to contribute to the optimization of therapeutic strategies for NAFLD.

## Materials and methods

### Search strategy and study selection

Three databases-PubMed, EMBASE, and the Cochrane Library-were searched without restrictions based on region, language, or publication type from the database’s inception until March 20, 2023. The search was performed with the Medical Subject Headings (MeSH) combined with free words: (NAFL OR NAFLD OR NASH OR "steatosis" OR "steatohepatitis" OR "fatty liver" OR "non-alcoholic fatty liver disease" OR "non-alcoholic steatohepatitis") AND (microbes OR microbiota OR microbiome OR flora OR microflora OR bacteria). The specific retrieval strategies are listed in S1 Table. A Population, Intervention, Comparator, Outcome and Study design (PICOS) scheme was used to clarify our research objectives. Representatives of the PICOS regimen are as follows: patients with NAFLD (P), comparison with healthy subjects (C), disturbances of the intestinal microbiota (O), and observational studies (S). Two researchers (Wenpin Cai and Ting Qiu) independently screened the titles and abstracts and carefully reviewed the full texts of potentially relevant articles, with any discrepancies resolved by a third researcher (Weitao Hu). Studies were included based on the following criteria (Table 1).

**Table 1:** Inclusion and exclusion criteria for NAFLD and controls.

Inclusion criteria	1. A confirmed diagnosis of NAFLD by ultrasound or histologic evidence
	2. Sequencing technology applied to analyze microbiota
	3. The alpha diversity index, or the relative or absolute abundance of microbial taxa was reported between NAFLD and healthy controls
	4. Normal liver function tests and no history of liver disease in the control group
Exclusion criteria	1. NAFLD patients with comorbidities of other liver diseases such as alcoholic fatty liver disease, viral hepatitis and autoimmune liver disease
	2. History of recent antibiotic use or other medications that affect gut microbes
	3. Animal studies or vitro studies
	4. Abstracts, case reports, expert opinions, reviews, letters or editorials
	5. Data not available or not convertible
	6. Without healthy controls

Abbreviations: NAFLD non-alcoholic fatty liver disease

### Outcomes

Outcomes included differences in gut microbiota abundance at the phylum and genus levels, as well as alpha diversity (including Shannon, Simpson, and Chao) between NAFLD and healthy controls.

### Data extraction and quality assessment

The following information was independently extracted from the included articles by two independent reviewers (Wenpin Cai and Ting Qiu) using a predesigned form: first author’s name, country, publication year, detection method, sample size, sex, age, body mass index (BMI), alanine aminotransferase (ALT), aspartate aminotransferase (AST), alpha diversity, and relative abundance of the gut microbiota. We focused on the statistical results of the gut microbial profiles and species diversity in the NAFLD and healthy groups. If necessary, we contacted the corresponding author to obtain or validate the information. Outcome indicators are expressed as mean ± standard deviation. The quality of the included studies was evaluated using the Newcastle-Ottawa Quality Rating Scale (NOS) [9]. The scale contains eight items, which are categorized into three groups: study group selection, group comparability (a maximum of two stars can be given for comparability), and exposure or outcome of interest determination for case-controlled or cohort studies. A star rating system was used to rate the quality of the included studies based on a scale ranging from 0 (low quality) to 9 (high quality). Any differences between the two researchers (Cai and Qiu) during the quality assessment process were resolved through consultation with a third researcher.

### Statistical analysis

Stata software version 17.0 was used for all statistical analyses in the present study. Pooled statistics for continuous data were expressed as standardized mean difference (SMD) and 95% confidence interval (CI), owing to the application of different methods to evaluate the same outcomes. The results of the analyses are presented as forest plots. The I^2^ statistic was used to assess the heterogeneity [10]. If the heterogeneity was relatively small (I^2^ < 50%), a fixed-effects model was used for analysis. Otherwise, a random-effects model was used. A sensitivity analysis was performed by excluding one study at a time to characterize the stability and accuracy of the studies. Publication bias [11] was assessed using funnel plots and Egger’s test. P < 0.05 was considered statistically significant difference.

## Results

### Literature search

**Fig 1:**
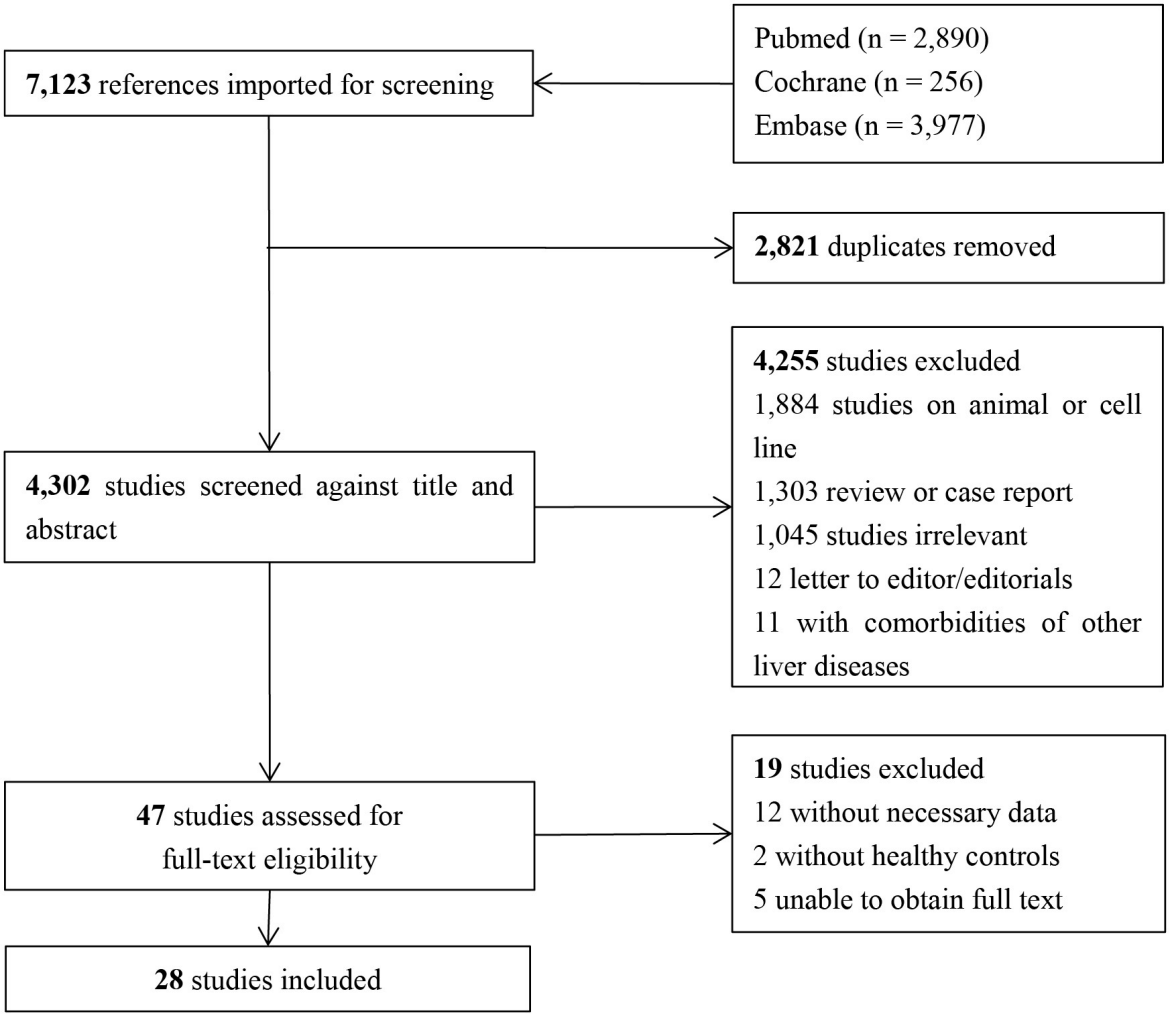
PRISMA flow diagram.

The literature screening process is illustrated in Fig 1. The literature was searched using three electronic databases (PubMed, 2890; Embase, 3977; and Cochrane Library, 256) with 7123 articles, and 28 articles were finally included.

### Study characteristics and quality assessment

A total of 28 articles [12–39] with 3566 participants were included in this study. The sequencing protocol was performed in all studies, of which 71.43% (20/28) [14, 16, 18–21, 23–27, 29–32, 34–38] involved 16S rRNA gene sequencing. Approximately 60.71% (17/28) [12–16, 18, 19, 22, 25, 26, 30, 31, 33, 35, 36, 38, 39] of the studies were conducted in Asia, 17.86% (5/28) [21, 23, 27, 29, 32] in Europe, 17.86% (5/28) [17, 20, 28, 34, 37] in North America, and only 3.57% (1/28) [24] in South America. Because only one study in South America was insufficient for subgroup analysis, studies other than those in Asia were uniformly categorized as studies from other continents. A quality assessment was performed, and all articles had a NOS score of 7 or higher. No studies were excluded because of poor NOS scores. The basic characteristics of the included studies and the specific quality assessment scores are listed in Tables 2 and 3, respectively.

**Table 2:** Characteristics of the included literature.

Study	Country	Study design	No. of case/control	Age		Sex (male/female)		BMI, kg/m^2^		ALT, U/L		AST, U/L		Sample	Microbiota detection method
				Case	Control	Case	Control	Case	Control	Case	Control	Case	Control		
Zhou et al., 2022 [12]	China	Case-control Study	27/31	14.00±2.06	13.87±1.65	21/6	21/10	30.40±3.37	24.63±4.72	NA	NA	NA	NA	feces	metagenomics
You et al., 2021 [13]	China	Case-control Study	79/32	41.9±12.5	38.03±11.49	43/36	15/17	26.25±1.22	21.71±0.45	53.07±13.88	18.77±3.39	30.87±6.78	18.47±1.39	feces	internal transcribed spacer sequencing
Yang et al., 2022 [14]	China	Case-control Study	20/19	49.15±3.01	55.37±2.43	14/6	7/12	26.60±0.73	26.60±0.73	41.33±6.32	13.24 ±1.29	27.67±2.58	17.28±1.02	feces	16S rRNA gene sequencing
Yang et al., 2022 [15]	China	Case-control Study	32/30	41.26±14.55	40±14.6	26/6	18/12	26.21±3.80	26.21±3.80	69.27±40.94	19.25±10.51	35.95±18.24	19.77±4.09	feces	16S rDNA sequencing
Vernekar et al., 2018 [16]	India	Cross-sectional study	11/9	40.14±6.72	36.44±7.51	6/5	7/2	27.2±1.45	23.76±0.43	62.10±12.42	34.83±5.79	60.45±14.2	60.45±14.2	feces	16S rRNA gene sequencing
Testerman et al., 2022 [17]	USA	Cross-sectional study	18/18	12.54± 2.42	12.22±3.02	11/ 7	8/10	35.21±6.82	30.26±7.04	40.14±33.80	18.33±7.27	32.06±26.93	20.83± 4.99	feces	shotgun metagenome sequencing
Si et al., 2021 [18]	Korea	Case-control Study	187/41	49.98±14.53	58.34±10.53	98/89	64/50	28.22±3.85	23.43±2.45	68.36±60.10	27.46±24.74	49.34±41.23	29.66±18.53	feces	16S rRNA gene sequencing
Shi et al., 2021 [19]	China	Cross-sectional study	421/1138	68.36±7.9	68.9±9.16	188/233	571/567	25.97±3.39	23.7±3.41	NA	NA	NA	NA	feces	16S rRNA gene sequencing
Schwimmer et al., 2019 [20]	USA	Cross-sectional study	87/37	12±3.02	12.64±2.31	62/25	17/20	29.7±6.03	28.29±4.63	64.75±48.25	16±6.17	41.06±21.86	21.07±5.4	feces	16S rRNA gene sequencing
Rau et al., 2018 [21]	Germany	Case-control Study	32/27	48.84±13.35	27.0±3.3	13/19	8/19	46.54±9.41	21.4±2.9	NA	NA	NA	NA	feces	16S rRNA gene sequencing
Asaji et al., 2022 [22]	Japan	Case-control Study	7/7	63.6±8.4	64.7±7.7	5/2	4/3	25.1±2.8	21.8±1.6	42±13	16±6	36±11	19±4	intestinal mucus, feces	16S rDNA sequencing
Demir et al., 2020 [23]	Germany	Cross-sectional study	90/21	50.5±24.8	38±25.0	52/38	5/16	29.9±6.4	20±1.3	50.5±46.5	13±11.5	33.5±22.8	24.5±7.8	feces	16S rRNA gene sequencing
Kordy et al., 2021 [24]	Brazil	Cross-sectional study	21/54	13.43±3.44	12.08±3.3	14/7	24/30	34.04±7.99	18.27±2.38	231.63±254.83	26.59±5.06	141.34±163.01	32.75±6.82	feces	16S rRNA gene sequencing
Pan et al., 2021 [25]	China	Case-control Study	50/25	10.88±2.16	10.08±1.78	46/4	23/2	28.11±3.97	28.21±1.32	67.17±45.33	27.63±13.46	35.61±18.50	22.61±11.37	feces	16S rRNA gene sequencing
Oh et al., 2021 [26]	Korea	Case-control Study	22/44	46.18±19.73	51±6.13	6/16	12/32	28.7±3.33	21.19±1.76	94.52±57.85	16.21±5.36	71±50.32	20.11±4.83	feces	16S rRNA gene sequencing
Kravetz et al., 2020 [27]	Italy	Cross-sectional study	44/29	13.3±3.2	12.9 ±2.8	25/19	13/16	35.5±6.4	32.5±6.8	46.5±32.2	17.9±7.6	NA	NA	feces	16S rRNA gene sequencing
Moran-Ramos et al., 2023 [28]	Mexico	Cross-sectional study	23/11	36.55±3.63	38.41±7.53	7/16	2/9	43.24±2.51	43.09±4.61	46.51±10.62	23.58±8.15	36.17±4.53	25.36±2.60	feces	whole genome shotgun sequencing
Chierico et al., 2017 [29]	Italy	Case-control Study	53/54	12.15±2.61	10.24±2.51	32/21	23/31	26.93±5.48	17.59±1.79	38.26±20.75	NA	30.79±15.17	NA	feces	16S rRNA gene sequencing
Jiang et al., 2015 [30]	China	Cross-sectional study	53/32	47.83±11.03	40.54±6.29	26/27	5/27	26.56±2.60	23.26±3.70	48.67±29.57	20.19±5.56	34.74±20.08	20.69±5.32	feces	16S rRNA gene sequencing
Li et al., 2018 [31]	China	Case-control Study	30/37	47.53±8.51	44.24±9.19	15/15	11/26	27.19±2.56	23.37±2.21	27.0±17.63	16.7±8.51	19.9±5.96	17.5±3.91	feces	16S rRNA gene sequencing
Nistal et al., 2019 [32]	Spain	Case-control Study	36/20	49.23±7.80	39.16±8.83	14/22	7/13	46.57±5.24	21.13±3.39	38.23±17.97	22.29±5.88	29.61±17.49	20.64±4.54	feces	16S rRNA gene sequencing
Shen et al., 2017 [33]	China	Case-control Study	25/22	45.5±10.1	45.5±10.1	19/6	17/5	28.6±3.5	21.6±1.7	51.6±34.5	17.7±5.3	26.9±12.2	20.6±6.6	feces	16S rDNA sequencing
Silva et al., 2018 [37]	Canada	Cross-sectional study	39/28	47.40±8.86	37.24±9.2	20/19	15/13	31.93±6.49	26.8±3.93	67.17±36.30	19.11±8.45	42.30±22.89	19.75±4.23	feces	16S rRNA gene sequencing
Sobhonslidsuk et al., 2018 [36]	Thailand	Case-control Study	16/8	59.8±9.6	43.4±6.8	13/3	8/0	27.7±4.8	21.3±1.2	59±30	17±6	49.7±11.9	24.4±11.6	feces	16S rRNA gene sequencing
Wang et al., 2016 [39]	China	Cross-sectional study	10/15	42.5±8.32	37.9±8.64	7/3	10/5	22.3±3.48	20.8±2.71	28.0±11.99	12.0±5.62	21.0±6.01	17.0±4.69	feces	pyrosequencing
Wong et al., 2013 [38]	Hong Kong, China	longitudinal study	16/22	51±9	44±10	9/7	9/13	29.1±5.6	22.2±2.7	72.03±40.64	23.08±10.30	38.55±19.51	20.36±5.55	feces	16S rRNA gene sequencing
Yun et al., 2019 [35]	Korea	Cross-sectional study	76/192	45.3±8.2	42.9±8.2	55/21	83/109	25.7±2.6	22.2±2.4	24.5±12.9	20.9±17.9	22.1±7.3	19.1±5.5	feces and blood	16S rRNA gene sequencing
Zhu et al., 2013 [34]	America	Case-control Study	22/16	13.6±16.42	14.4±7.2	12/10	10/6	34±1.88	20.4±0.4	66.9±8.91	NA	51.7±6.1	NA	feces	16S rRNA gene sequencing

Abbreviations: BMI body mass index, ALT alanine aminotransferase, AST aspartate aminotransferase, NA not available

**Table 3:** Quality assessment of included studies by means of the Newcastle-Ottawa Scale.

study	Case definition	Representativeness	Selection of controls	Definition of controls	Comparability	Ascertainment of	Same method	nonresponse	score
Zhou et al., 2022 [12]	1	1	0	1	1	1	1	1	7
You et al., 2021 [13]	1	1	1	1	2	1	1	1	9
Yang et al., 2022 [14]	1	1	1	1	0	1	1	1	7
Yang et al., 2022 [15]	1	1	1	1	2	1	1	1	9
Vernekar et al., 2018 [16]	1	1	0	1	1	1	1	1	7
Testerman et al., 2022 [17]	1	0	0	1	2	1	1	1	7
Si et al., 2021 [18]	1	1	1	1	0	1	1	1	7
Shi et al., 2021 [19]	1	1	1	1	1	1	1	1	8
Schwimmer et al., 2019 [20]	1	1	0	1	2	1	1	1	8
Rau et al., 2018 [21]	1	1	0	1	1	1	1	1	7
Asaji et al., 2022 [22]	1	1	0	1	2	1	1	1	8
Demir et al., 2020 [23]	1	1	1	1	0	1	1	1	7
Kordy et al., 2021 [24]	1	0	0	1	2	1	1	1	7
Pan et al., 2021 [25]	1	1	0	1	2	1	1	1	8
Oh et al., 2021 [26]	1	1	1	1	2	1	1	1	9
Kravetz et al., 2020 [27]	1	1	0	1	2	1	1	1	8
Moran-Ramos et al., 2023 [28]	1	0	0	1	2	1	1	1	7
Chierico et al., 2017 [29]	1	1	0	1	1	1	1	1	7
Jiang et al., 2015 [30]	1	1	1	1	1	1	1	1	8
Li et al., 2018 [31]	1	1	0	1	2	1	1	1	8
Nistal et al., 2019 [32]	1	1	1	1	1	1	1	1	8
Shen et al., 2017 [33]	1	1	1	1	2	1	1	1	9
Silva et al., 2018 [37]	1	1	1	1	1	1	1	1	8
Sobhonslidsuk et al., 2018 [36]	1	1	1	1	1	1	1	1	8
Wang et al., 2016 [39]	1	0	1	1	2	1	1	1	8
Wong et al., 2013 [38]	1	1	1	1	2	1	1	1	9
Yun et al., 2019 [35]	1	1	1	1	1	1	1	1	8
Zhu et al., 2013 [34]	1	1	1	1	2	1	1	1	9

### Alpha diversity

**Fig 2:**
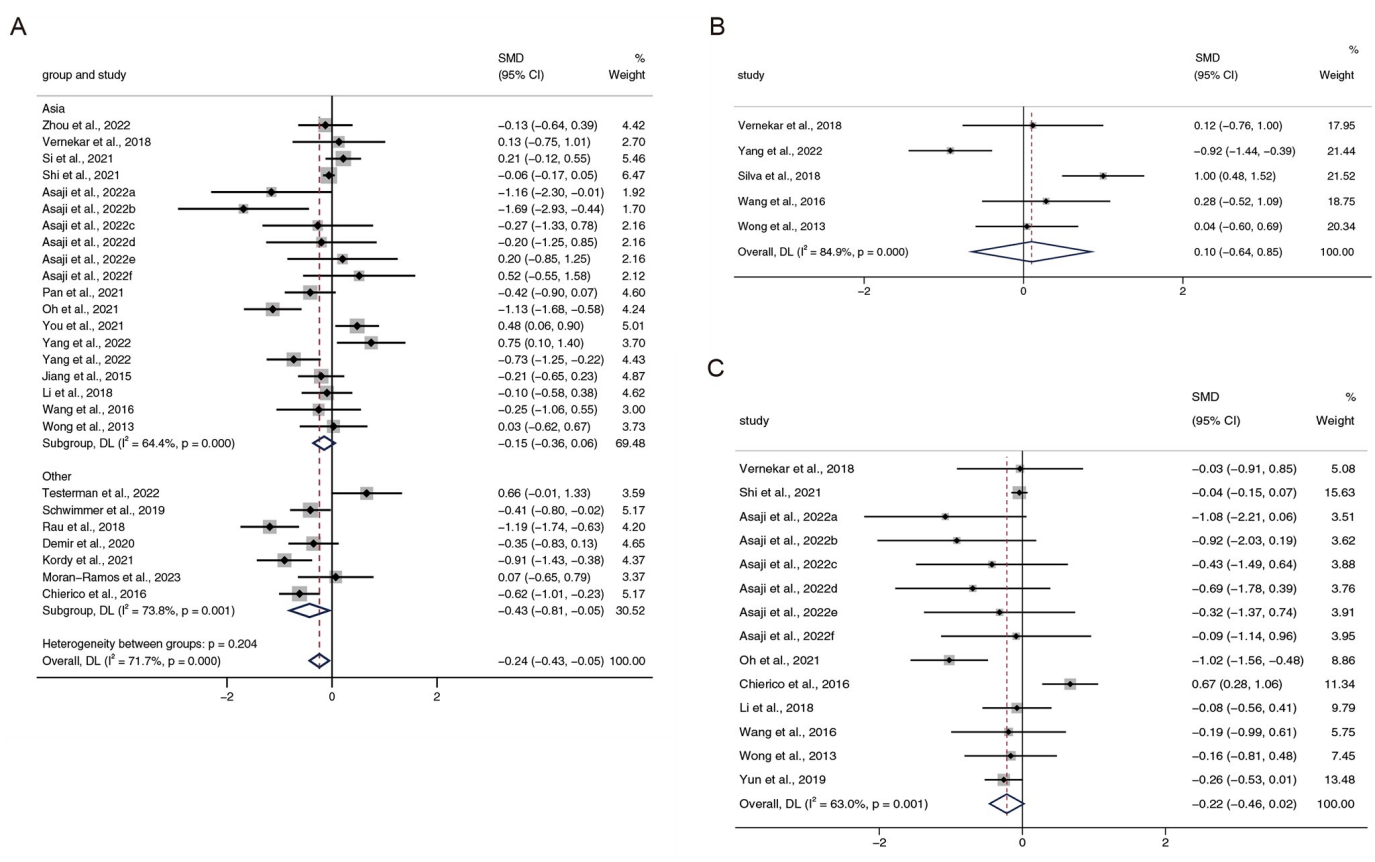
Alpha diversity outcomes. Forest plots for (A) Shannon index, (B) Simpson, (C) Chao. a: faecal samples; b: terminal ileum biopsy samples; c: cecum biopsy samples; d: transverse colon biopsy samples; e: sigmoid colon biopsy samples; f: rectum biopsy samples.

Alpha diversity provides a prediction of species richness and evenness and is measurable by the Shannon, Simpson, and Chao indices. A total of 21 papers reported the Shannon index [13, 14, 18, 21, 22, 26, 29–31, 34, 39]. Among them, Asaji [22] et al. collected samples that contained mucus from different intestinal segments (including the terminal ileum, cecum, transverse colon, sigmoid colon, and rectum), in addition to feces. All data met the inclusion criteria and hence were included. Shannon diversity was reduced in patients with NAFLD compared to that in normal subjects (SMD = -0.24, 95% CI -0.43, -0.05, I^2^ = 71.7%). Considering the high heterogeneity, studies were categorized into Asia and other continents based on geography. Findings from the subgroup analyses demonstrated a decreasing trend in the Shannon index both in Asia (SMD = -0.15, 95% CI -0.36, -0.06, I^2^ = 64.4%) and other continents (SMD = -0.43, 95% CI -0.81, -0.05, I^2^ = 73.8%), although the reduction was not significant in Asia. The SMDs for Simpson [15, 16, 37–39] and Chao [16, 19, 22, 26, 29, 35, 38, 39] were 0.10 (95% CI -0.64 to 0.85, I^2^ = 84.9%) and -0.22 (95% CI -0.46 to 0.02, I^2^ = 63.0%), respectively, suggesting that there were no obvious distinctions between NAFLD and healthy individuals (Fig 2).

### The different abundance of microbiota at the phylum level

**Fig 3:**
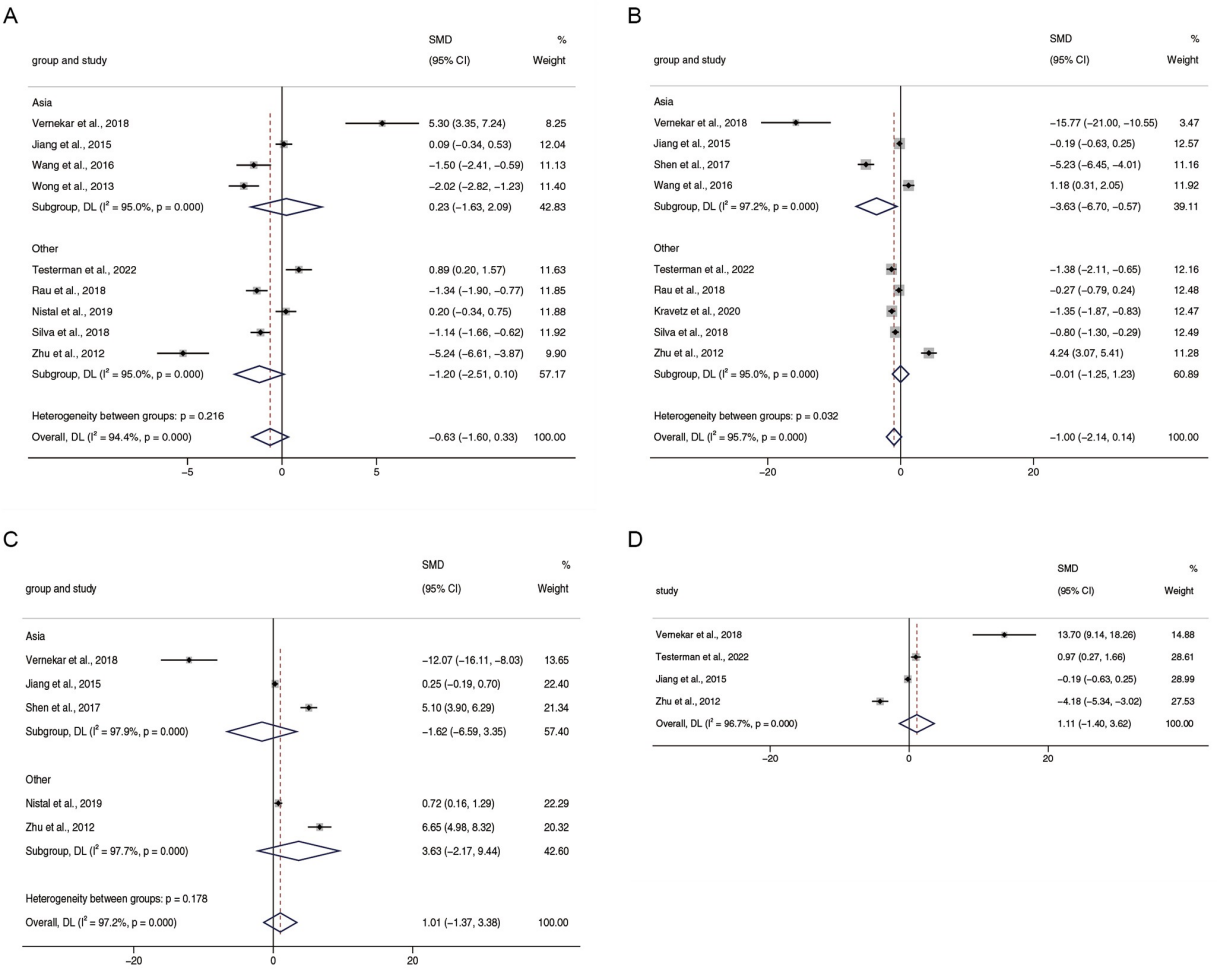
Forest plots for relative abundance of phyla (A) *Firmicutes*, (B) *Bacteroidetes*, (C) *Proteobacteria*, and (D) *Actinobacteria*.

The relative abundance of *Firmicutes* (SMD = -0.63, 95% CI -1.00 to 0.33, I^2^ = 94.4%) was reported in 9 literatures [16, 17, 21, 30, 32, 34, 37–39], suggesting an absence of significant differences in NAFLD and healthy individuals. The results of subgroup analyses were consistent. The relative abundance of *Bacteroidetes* was reported in 9 studies [16, 17, 21, 27, 30, 33, 34, 37, 39], and its total SMD was -1.00 (95% CI -2.14 to 0.14, I^2^ = 95.7%), implying that NAFLD caused no significant effect on it. Interestingly, the results of subgroup analysis showed that the relative abundance of *Bacteroidetes* was lower in NAFLD patients as compared to healthy individuals in Asia (SMD = -3.63, 95% CI -6.70 to -0.57, I^2^ = 97.2%). In contrast, no differences were observed in other continental regions (SMD = -0.01, 95% CI -1.25 to 1.23, I^2^ = 95.0%). Summarizing the 5 articles [16, 30, 32–34], the total *Proteobacteria* SMD was 1.01 (95% CI -1.37 to 3.38, I^2^ = 97.2%), showing that NAFLD did not influence the relative abundance of *Proteobacteria*. No changes were observed in the subgroup analysis. Only 4 papers reported *Actinobacteria* [16, 17, 30, 34] with an SMD of 1.11 (95% CI -1.40 to 3.62, I^2^ = 96.7%), which is highly heterogeneous (Fig 3).

### The different abundance of microbiota at the genus level

**Fig 4:**
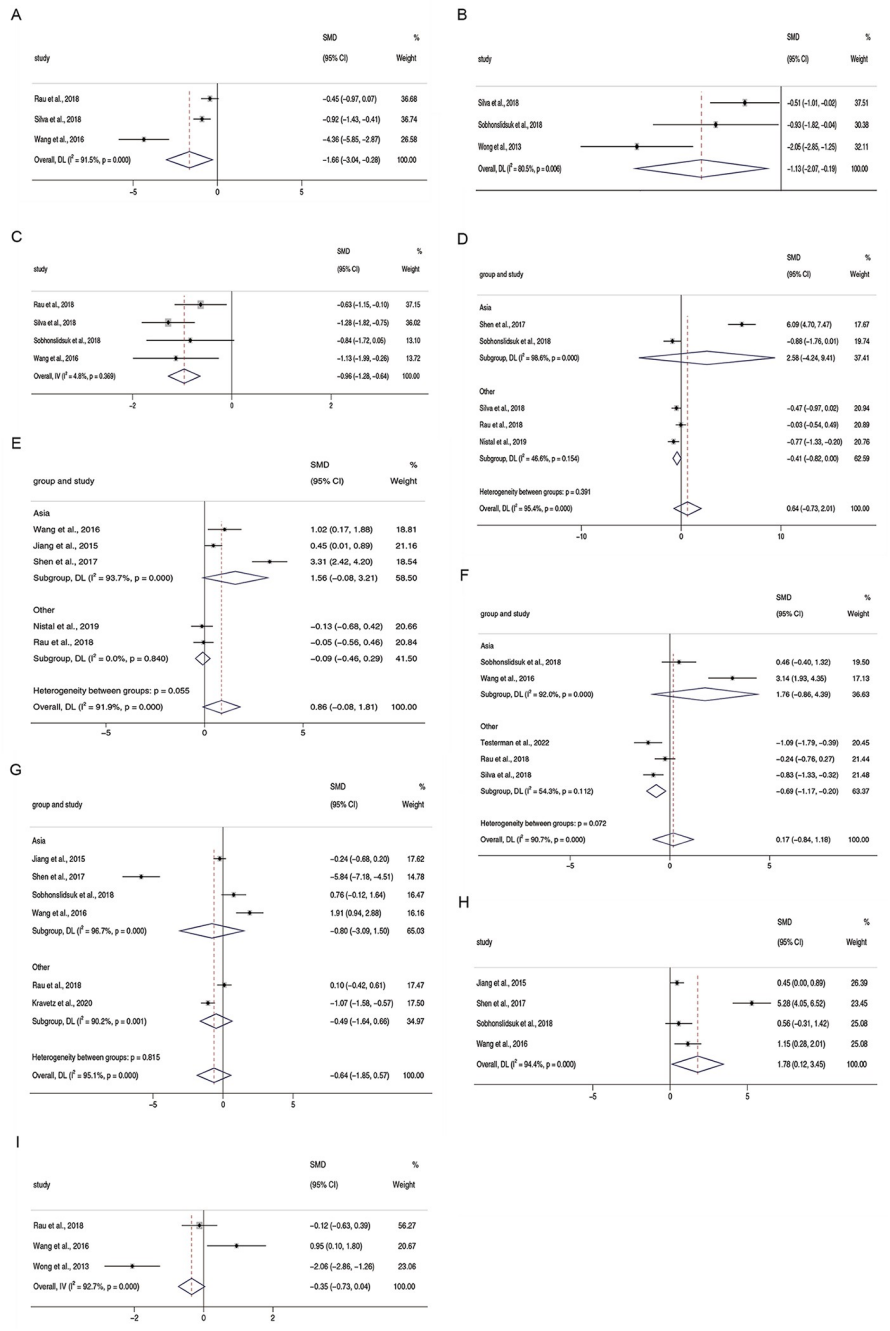
Forest plots for the relative abundance of genera (A) *Coprococcus*, (B) *Faecalibacterium*, (C) *Ruminococcu*s, (D) *Blautia*, (E) *Streptococcus*, (F) *Bacteroides*, (G) *Prevotella*, (H) *Escherichia*, and (I) *Bifidobacterium*.

The total SMDs for *Coprococcus* [21, 37, 39], *Faecalibacterium* [36–38], and *Ruminococcus* [21, 36, 37, 39] were -0.96 (95% CI -1.29 to -0.63, I^2^ = 4.8%), -1.13 (95% CI -2.07 to -0.19, I^2^ = 80.5%), and -1.66 (95% CI -3.04 to -0.28, I^2^ = 91.5%), suggesting that these genera are lacking in NAFLD patients. Both the total SMD (SMD = 0.64, 95% CI -0.73 to 2.01, I^2^ = 95.4%) and the results of subgroup analyses showed that the relative abundance of *Blautia* [21, 32, 33, 36, 37] was not altered in NAFLD. The relative abundance of *Streptococcus* (SMD = 0.86, 95% CI -0.08 to 1.81, I^2^ = 91.9%) [21, 30, 32, 33, 39] was also not affected by NAFLD. The relative abundance of *Bacteroides* (SMD = 0.17, 95% CI -0.84 to 1.18, I^2^ = 90.7%) [17, 21, 36, 37, 39] was not linked to NAFLD. Nevertheless, it is noteworthy that after subgroup analysis of different geographic regions, the result of 3 studies from the United States, Germany, and Canada revealed a decrease in the relative abundance of *Bacteroides* (SMD = -0.69, 95% CI -0.17 to -0.20, I^2^ = 54.3%) in the presence of NAFLD. *Prevotella* (SMD = -0.64, 95% CI -1.85 to 0.57, I^2^ = 95.1%) [21, 27, 30, 33, 36, 39] exhibited no significant difference in NAFLD versus controls. A total of 4 papers reporting on *Escherichia* [30, 33, 36, 39] demonstrated an increase in its relative abundance in NAFLD compared to healthy individuals (SMD = 1.78, 95% CI 0.12 to 3.45, I^2^ = 94.4%). Based on data from three publications describing *Bifidobacterium* [21, 38, 39], no difference in the relative abundance between NAFLD and healthy individuals was found (SMD = -0.35, 95% CI -0.73, 0.04, I^2^ = 92.7%) (Fig 4).

### Evaluation for publication bias

**Fig 5:**
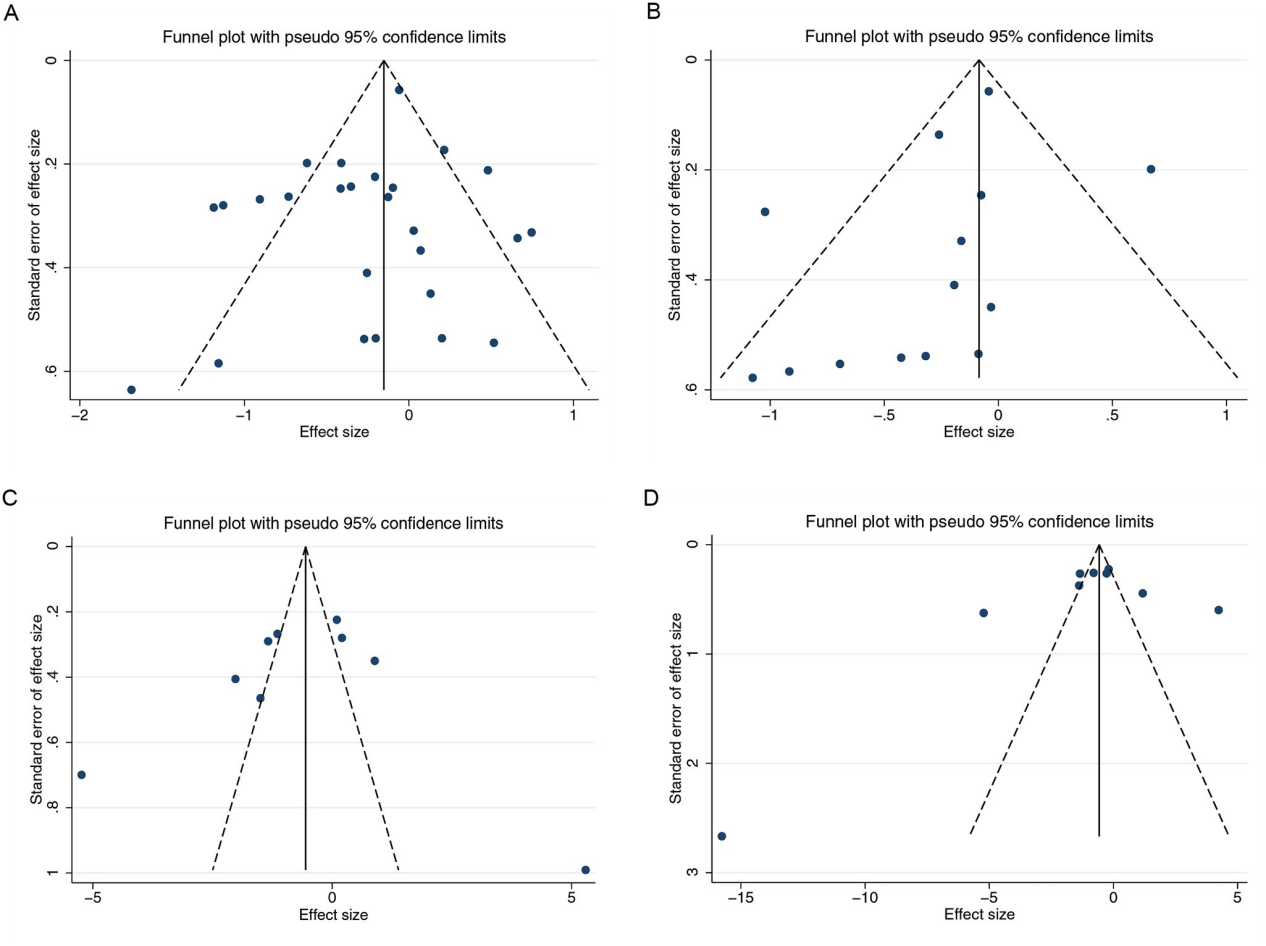
Publication bias detection. Funnel plots for (A) Shannon, (B) Chao, (C) *Firmicutes*, and (D) *Bacteroidetes*.

Due to the high number of studies (n ≥ 9) of Shannon, Chao, *Bacteroidetes*, and *Firmicutes*, funnel plots were constructed, and Egger’s test was performed to evaluate the existence of publication bias. The funnel plots of Chao and *Bacteroidetes* were asymmetric, but the Egger’s p-values were greater than 0.05, indicating no significant publication bias (P = 0.692; P = 0.526). The funnel plots of the Shannon and *Firmicutes* indices were symmetrical, and Egger’s test did not show any evidence of publication bias (P = 0.251; P = 0.749) (Fig 5).

### Sensitivity analysis

**Fig 6:**
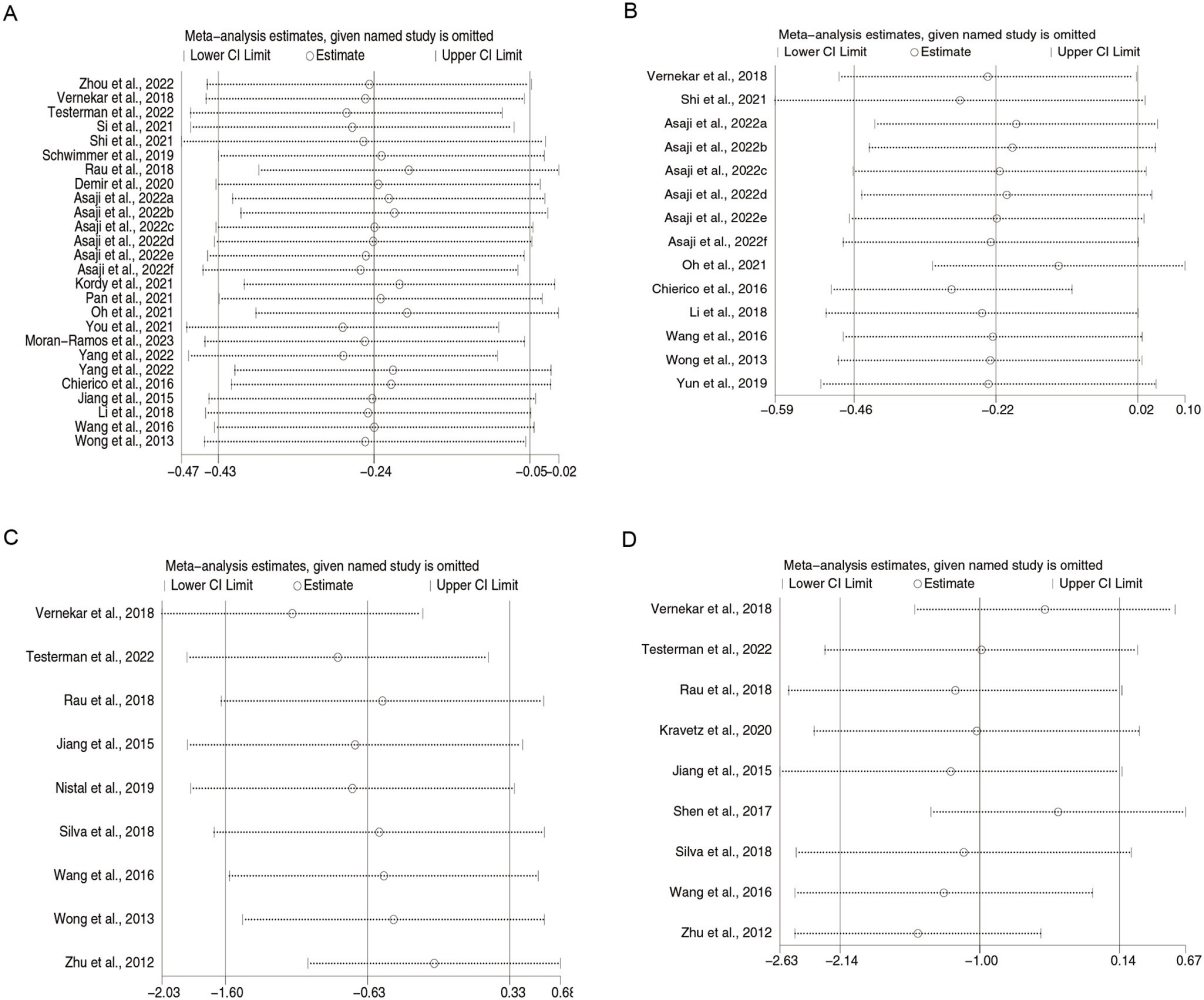
Sensitivity analysis of (A) Shannon, (B) Chao, (C) *Firmicutes*, and (D) *Bacteroidetes*.

Sensitivity analysis for Shannon, Chao, *Firmicutes*, and *Bacteroidetes* with item-by-item exclusion using Stata software revealed that all results remained unaltered, suggesting that the included studies were stable (Fig 6).

## Discussion

There is growing evidence for a link between perturbations in the intestinal microbiota and the pathophysiology of NAFLD, and some of these alterations can contribute to the evolution of the disease [37, 40, 41]. Therefore, identifying specific alterations in gut microbiota in NAFLD is crucial.

In the updated meta-analysis, with 28 articles from 13 countries involving 3566 subjects, we systematically reviewed studies of gut microbiota in NAFLD and healthy individuals in terms of alpha diversity and relative abundance of different taxa. In cases in which a sufficient number of studies reported results, subgroup meta-analyses were used to check for heterogeneity in the geographic cohort. Alpha diversity represents the richness and homogeneity of the gut microbiota. Compared with the healthy population, the Shannon index was significantly decreased in patients with NAFLD, whereas studies in Asia showed only a small and non-significant reduction. Neither the Simpson nor the Chao indices demonstrated meaningful alterations, but subgroup analyses revealed that Chao levels significantly declined in Asian patients with NAFLD. Our meta-analysis suggests that species richness is reduced in individuals with NAFLD. A greater microbiome abundance and diversity generally indicates a healthier gut microbial ecosystem [42] and a more substantial physical condition. The decreased diversity of the intestinal microbiota in patients with NAFLD indicates a disruption in the microecological stability of the microbiota.

This study found insignificant changes in the gut microbiota at the phylum level. The relative abundance of *Firmicutes* and *Bacteroidetes* showed only small, unremarkable decreases in patients with NAFLD. *Proteobacteria* and *Actinobacteria* increased slightly, but not dramatically. Geographical stratification revealed that Asian patients had significantly lower levels of *Bacteroidetes* than healthy controls. *Escherichia* were markedly elevated at the genus level in patients with NAFLD, whereas *Coprococcus*, *Faecalibacterium*, and *Ruminococcus* declined significantly. The relative abundances of *Streptococcus*, *Bifidobacterium*, *Prevotella*, *Blautia*, and *Bacteroides* in patients with NAFLD were similar to those in healthy individuals.

It was revealed back in 2001 that NAFLD was linked to small intestinal bacterial overgrowth [43]. Short-chain fatty acids (SCFA) [44, 45], mainly acetate, propionate, and butyrate, are known to modulate host-gut microbiota interactions and protect against NAFLD. *Bacteroidetes* [46] are one of the largest groups of the gut microbiota, with many bacterial species capable of producing acetate (the most abundant SCFA). The decreased abundance of *Bacteroidetes* may have affected SCFA levels. *Coprococcus*, *Faecalibacterium*, and *Ruminococcus* contribute to the production of SCFA [47, 48]. *Coprococcus*, especially *Coprococcus eutactus*, was observed to reduce the concentration of pro-inflammatory cytokines TNF-α, IL-1β, and IL-6, as well as activate the protective effect of IgA selectively binding to pathogens, which resulted in the improvement of symptoms in mice with acute colitis [49]. *C*. *eutactus* promotes the generation of acetate [49], which has various beneficial functions in inhibiting inflammatory responses [50] and modulating insulin sensitivity [51] by binding to G-protein-coupled receptors [49]. A decreased abundance of *Coprococcus* may reduce intestinal SCFA levels and exacerbate NAFLD inflammation. The beneficial effects of *Faecalibacterium* appear to be associated with its anti-inflammatory properties [52]. *Faecalibacterium* strains fermenting glucose produce substantial amounts of butyrate [53, 54], which inhibits TNF-α-stimulated NF-κB activation in intestinal epithelial cells and blocks the production of IL-8 [52, 55]. Butyrate additionally suppresses histone deacetylase (HDAC), contributing to the expression of the dishevelled binding antagonist of beta-catenin 3 (*Dact3*), a gene encoding a negative regulator of the inflammatory Wnt/JNK signaling pathway, and represses the production of IL-8 [55, 56]. *Faecalibacterium* may play a role in NAFLD by affecting SCFA levels and the degree of inflammation. The genus *Ruminococcus* includes both beneficial and pro-inflammatory species. When consuming fruits and vegetables regularly, *Ruminococcus spp*. can ferment complex sugars to produce acetate, propionate, and butyrate, which have anti-inflammatory benefits [57]. Dietary intervention can effectively modify the microbiota and is one of the most important strategies for treating NAFLD. *Escherichia coli* is enriched in patients with NAFLD, especially in the advanced stages of fibrosis [58]. Ethanol generated by E. coli elevates intestinal permeability, followed by an increase in endotoxins from the intestinal lumen into the portal vein [59]. Endogenous ethanol and increased endotoxin levels may contribute to the development of NAFLD [59]. Modulating the intestinal microbiota, a complex microecosystem consisting of probiotics and pathogens, and restoring host-gut microbiota interactions may help in the fight against NAFLD.

The strengths of this study are that the data were generated based on sequencing, leading to high quality, and there was no restriction on the type of samples collected, covering the feces and mucosa of different anatomical sites. This study had some limitations. Considerable heterogeneity existed in the study results; however, the sources of heterogeneity in some of the outcome metrics could not be fully explained by subgroup and sensitivity analyses, but only by minimizing them as much as possible using random-effects models. The pathological progression of NAFLD may lead to changes in the structure of the gut microbiota, but subgroup analyses based on the NAFLD disease spectrum are not available temporally because of the small amount of data included. Subgroup analyses were stratified only for Asia and other continents, owing to the fact that some regions were too little studied. The subgroups should be more specific in the future with more new data collected, as dietary habits and lifestyles in different regions have a particular impact on the gut microbiota.

## Conclusion

In summary, NAFLD affects the richness and composition of the gut microbiota. It was found that the alpha diversity was reduced in patients with NAFLD. At the genus level, *Coprococcus*, *Faecalibacterium*, and *Ruminococcu*s decreased, whereas *Escherichia* was higher than that in healthy controls. After clarifying the changes in the characteristic microbial profile of NAFLD, modification of the diet structure and modality or development of new targeted drugs may be beneficial for the treatment of NAFLD.

## Supporting information

S1 Table Search strategies. (DOCX)

S1 Checklist (DOCX)
