# Supplementary material for: Changes in the intestinal microbiota of individuals with non-alcoholic fatty liver disease based on sequencing: An updated systematic review and meta-analysis
Source: PLoS One. 2024 Mar 28;19(3):e0299946. doi: 10.1371/journal.pone.0299946 (PMC10977702; doi:10.1371/journal.pone.0299946)
Supplement: S1 Table — (DOCX) [file pone.0299946.s001.docx]

Table S1: Search strategies

| PubMed | ("non-alcoholic fatty liver disease"[MeSH Terms] OR ("non-alcoholic fatty liver disease"[Title/Abstract] OR "NAFL"[Title/Abstract] OR "NAFLD"[Title/Abstract] OR "NASH"[Title/Abstract] OR "steatosis"[Title/Abstract] OR "steatohepatitis"[Title/Abstract] OR "fatty liver"[Title/Abstract] OR "non-alcoholic steatohepatitis"[Title/Abstract])) AND ("microbiota"[MeSH Terms] OR ("microbiota"[Title/Abstract] OR "microbes"[Title/Abstract] OR "microbiome"[Title/Abstract] OR "flora"[Title/Abstract] OR "microflora"[Title/Abstract] OR "bacteria"[Title/Abstract])) |
| --- | --- |
| Embase | #1 ‘non-alcoholic fatty liver disease’/exp  #2 ‘non-alcoholic fatty liver disease’:ab,ti OR ‘NAFL’:ab,ti OR ‘NAFLD’:ab,ti OR ‘NASH’:ab,ti OR ‘steatosis’:ab,ti OR ‘steatohepatitis’:ab,ti OR ‘fatty liver’:ab,ti OR ‘non-alcoholic steatohepatitis’:ab,ti  #3 ‘microbiota’/exp  #4 ‘microbiota’:ab,ti OR ‘microbes’:ab,ti OR ‘microbiome’:ab,ti OR ‘flora’:ab,ti OR ‘microflora’:ab,ti OR ‘microflora’:ab,ti  #5 #1 OR #2  #6 #3 OR #4  #7 #5 AND #6 |
| Cochrane | #1 MeSH descriptor: [Non-alcoholic Fatty Liver Disease] explode all trees  #2 (non-alcoholic fatty liver disease):ti,ab,kw OR (NAFL):ti,ab,kw OR (NAFLD):ti,ab,kw OR (NASH):ti,ab,kw OR (steatosis):ti,ab,kw  #3 (steatohepatitis):ti,ab,kw OR (fatty liver):ti,ab,kw OR (non-alcoholic steatohepatitis):ti,ab,kw  #4 MeSH descriptor: [Microbiota] explode all trees  #5 (microbiota):ti,ab,kw OR (microbes):ti,ab,kw OR (microbiome):ti,ab,kw  #6 (flora):ti,ab,kw OR (microflora):ti,ab,kw OR (bacteria):ti,ab,kw  #7 #1 OR #2 OR #3  #8 #4 OR #5 OR #6  #9 #7 AND #8 |
